# The association of serum lipids with the histological pattern of rectosigmoid adenoma in Taiwanese adults

**DOI:** 10.1186/1471-230X-11-54

**Published:** 2011-05-15

**Authors:** Zih-Jie Sun, Ying-Hsiang Huang, Jin-Shang Wu, Yi-Ching Yang, Ying-Fang Chang, Feng-Hwa Lu, Chih-Jen Chang

**Affiliations:** 1 Department of Family Medicine, National Cheng Kung University College of Medicine and Hospital, Dou-Liou Branch, No.345, Zhuangjing Rd., Douliou City, Yunlin County 640, Taiwan; 2 Department of Family Medicine, National Cheng Kung University Hospital, No.138, Shengli Rd., East Dist., Tainan City 701, Taiwan

## Abstract

**Background:**

The mortality rate of colorectal cancer ranks third behind lung and hepatic cancer in Taiwan. Colorectal cancer mostly arises from adenomatous polyps of left colon. The aim of our study was to examine the association of serum lipids with the histological pattern of rectosigmoid adenoma.

**Methods:**

There were 2,506 eligible examinees aged 20 and above who underwent sigmoidoscopy as a screening examination in National Cheng Kung University Hospital between January 2003 and October 2006. They were classified into three groups: tubular adenoma (333 subjects), villous-rich (tubulovillous/villous) adenoma (53 subjects) and normal (2,120 subjects). We defined high total cholesterol (TC) as a level ≧200 mg/dl, low high-density lipoprotein cholesterol (HDL-C) as a level <40 mg/dL, and high triglyceride (TG) as a level ≧200 mg/dl according to the third report of the National Cholesterol Education Program expert panel on detection, evaluation, and treatment of high blood cholesterol in adults. Adenoma histology was classified as tubular, tubulovillous and villous according to the proportion of villous part.

**Results:**

Among the study population, 333 subjects (13.3%) had tubular adenomas and 53 subjects (2.1%) had villous-rich adenomas. The odds ratio (OR) for villous-rich adenoma in subjects with TG≧200 mg/dL compared to those with TG < 200 mg/dL was 3.20 (95% confidence interval [CI]:1.71-6.01), after adjusting for age, gender, general obesity, central obesity, diabetes, hypertension, smoking, and alcohol consumption. If further taking high TC and low HDL-C into consideration, the OR was 4.42 (95% CI:2.03-9.63).

**Conclusions:**

Our study showed that subjects with high serum TG tended to have a higher risk of tubulovillous/villous adenoma in rectosigmoid colon. Therefore, reducing the serum TG level might be one method to prevent the incidence of colorectal cancer.

## Background

According to a report of the World Health Organization, cancer was the leading cause of death in 2007, accounting for 7.9 million deaths, or 13% of the total amount. The same report stated that colorectal cancer was the fourth most common fatal cancer, after lung, stomach, and liver cancer [1]. In addition, the Department of Health in Taiwan indicated that cancer was the major cause of death from 1986 to 2008, with colorectal cancer ranking third, after lung and liver cancer [2].

Seventy percent of colorectal cancer cases occur in the left colon, including rectum, sigmoid and partial descending colon [3], and colorectal cancer usually develops from colorectal polyps, especially adenomatous polyps [4]. According to histological patterns, adenoma types are classified into tubular, tubulovillous, and villous, with tubular adenoma being the most common, and villous adenoma being the least. Tubular adenoma has a 4% risk of developing malignancy, while tubulovillous and villous adenomas may have risks up to 40% [5]. Therefore, it is important to understand the factors influencing colorectal adenoma and its histology.

Previous studies on the relationship between serum lipids and colorectal adenoma show conflicting results. Serum triglyceride [6-9] and cholesterol [10-12] level are positively related to an increased risk of colorectal adenoma in some studies, while several investigators report an insignificant or even inverse relationship between serum lipids and colorectal adenoma [13-15]. These inconsistent results might be partially due to the association of serum lipids with different histological types of colorectal adenoma, although such topic has received little attention [16,17]. Therefore, we examined the association of serum lipids with the histology of rectosigmoid adenoma, hoping to provide useful information for preventing colorectal cancer.

## Methods

### Subjects

This is a retrospective research in which study subjects were selected from 4,844 examinees aged 20 or above who finished a health checkup with sigmoidoscopy as a screening examination at the health promotion center of National Cheng Kung University Hospital between January 2003 and October 2006. We excluded subjects with the following conditions: using medication for hyperlipidemia; past history of cancer, inflammatory bowel disease, familial adenomatous polyposis or thyroid disease; major gastrointestinal surgery, including partial or total gastrectomy or colorectomy; colon cancer diagnosed during sigmoidoscopic examination; vegetarian; dieting; liver cirrhosis or SGPT levels three times higher than the normal limit; nephrotic syndrome or serum creatinine levels higher than 1.5 mg/dL; CEA levels higher than 10 ng/mL; incomplete examination and missing data. Finally, 2,506 eligible subjects were drawn from the original examinees.

### Study Design

Each examinee was interviewed and received physical examination by family physicians, and also completed a structured questionnaire. The questionnaire gathered basic personal information (age, gender, education level, occupation, and marital status), personal and family medical history (including details of hypertension, diabetes mellitus, cardiovascular disease, cerebrovascular disease, and cancer.), dietary habits, and lifestyle (including cigarette smoking and alcohol consumption).

The examinees fasted for at least eight hours, and also stopped smoking, drinking alcohol, tea or coffee, and taking personal medication. Intravenous blood was collected and immediately sent to a central laboratory for analysis of the complete blood count, lipid profile, fasting glucose, uric acid, HbA1c, liver function test, renal function test, electrolytes, and so on. All subjects, except pregnant women or diabetic patients with oral hypoglycemic agents or insulin injection, received a 75-gram oral glucose tolerance test and were checked for post-load two-hour blood glucose. Diabetes mellitus was diagnosed according to the American Diabetes Association diagnostic criteria [18]. Blood pressure was measured with a DIANAMP vital sign monitor [Model 1846SX DINAMAP Monitor, Critikon Inc., USA) after five minutes of supine rest in a quiet examination room. Hypertension was defined as a systolic blood pressure (SBP)≧140 mmHg or diastolic blood pressure (DBP)≧90 mmHg according to the Seventh Report of the Joint National Committee [19], or when the subjects had a history of hypertension, or were receiving antihypertensive treatment.

Total cholesterol and triglyceride levels were measured by an enzymatic colorimetric test, and high density lipoprotein cholesterol was measured by a direct method in an automatic biochemical analyzer (Model 7600, Hoffmann-La Roche Inc., USA). According to the third report of the National Cholesterol Education Program expert panel on detection, evaluation and treatment of high blood cholesterol in adults (NCEP-ATP III), total cholesterol (TC) ≧240 mg/dL was defined as high, high-density lipoprotein cholesterol (HDL-C) levels <40 mg/dL as low and triglyceride (TG) levels ≧200 mg/dL as high [20].

Anthropometric measurements were performed by well-trained technicians and nurses. Body weight and height were measured with an electronic scale (Model HM-586, Jeng Jyi Co. Ltd, TAIWAN). Body mass index (BMI) was calculated with weight (in kilograms) divided by height (in meters) squared. Waist circumference was measured at the level of the umbilicus and hip circumference at the largest circumference over the buttocks with a tape measure while standing relaxed. BMI≧25 kg/m^2 ^is defined as general obesity and waist circumference ≥90 cm in men or ≥80 cm in women is defined as abdominal obesity, according to Asia-Pacific criteria of World Health Organization [21].

Experienced gastroenterologists used flexible sigmoidscopes (Model ES-200ER Fujinon Inc., JAPAN) to examine all subjects at least 60 centimeters from the anus. When polyps were found in the colon or rectum, a biopsy was conducted and sent to experienced pathologists for interpretation. Colorectal adenomas were classified into three subtypes according to the percentage of tubular vs. villous architecture. Specifically, tubular adenomas exhibit more than 75% tubular architecture, villous adenomas have more than 50% of villous architecture, and tubulovillous adenomas contain 25 to 50% villous architecture [22].

### Statistical analysis

We used SPSS statistical software version 12.0 for data analyses. Study subjects were first classified into two groups: normal and adenoma. The later was further classified into tubular adenomas and villous-rich (tubulovillous/villous) adenoma. Group differences for continuous variables were assessed by independent t-test or one way ANOVA, and the Chi-square test was used for categorical variables. Multinomial logistic regression was performed to calculate the odds ratio (OR) and 95% confidence interval (CI) of lipid profiles on the risk of tubular adenoma and villous-rich adenoma. We adjusted for age and gender in Model 1, and further adjusted for general obesity, abdominal obesity, diabetes, hypertension, smoking, and alcohol consumption in Model 2. Finally, high TC, high TG and low HDL-C were further added in Model 3. A p value < 0.05 was selected for statistical significance.

All authors declare that this research has followed all applicable institutional and governmental regulations concerning ethics and has been approved by the Institutional Review Board of National Cheng Kung University Hospital in Taiwan.

## Results

A total of 2,506 subjects were used in the analysis. There were 995 women (39.7%) and 1,511 men (60.3%) with a mean age of 50.6 ± 12.4 (20.2-88.6 years). Among the study population, 333 subjects (13.3%) had tubular adenomas and 53 subjects (2.1%) had villous-rich adenomas. Table 1 shows demographic and clinical characteristics of normal and adenoma groups. Adenoma group was older, and had higher fasting glucose, TC, TG and blood pressure, and lower HDL-C levels than normal subjects. Male gender, diabetes, hypertension, general obesity, abdominal obesity, smoking, and alcohol consumption were also more common in adenoma group.

**Table 1:** Comparisons of demographic and clinical characteristics between normal and adenoma groups

Variables	Normal (n = 2120)	Adenoma (n = 386)	P * value
Age (years)	49.6 ± 12.4	56.0 ±10.8	<0.001
Male, N (%)	1239 (58.4%)	272(70.5%)	<0.001
BMI (kg/m^2^)	24.5 ± 3.4	25.2 ±3.4	<0.001
WC (cm)	84.3 ± 10.4	87.5 ±10.4	<0.001
Fasting glucose (mg/dL)	93.3 ± 22.8	100.9 ±28.3	<0.001
Total cholesterol (mg/dL)	194.7 ± 36.9	200.3 ±38.5	0.007
Triglyceride(mg/dL)	129.4 ± 79.2	146.0 ±83.7	<0.001
HDL-C (mg/dL)	45.5 ± 11.8	43.7 ±11.8	0.005
SBP (mmHg)	117.4 ± 20.4	122.6 ±17.2	<0.001
DBP (mmHg)	68.8 ± 13.9	72.0 ±10.0	<0.001
General obesity^‡^, N (%)	869 (41.0%)	194(50.3%)	0.001
Abdominal obesity^§^, N (%)	894 (42.2%)	202(52.3%)	<0.001
Hypertension, N (%)	478 (22.5%)	124(32.1%)	<0.001
Diabetes mellitus, N (%)	304 (14.3%)	99(25.6%)	<0.001
Smoking, N (%)	616 (29.1%)	144(37.3%)	0.001
Alcohol consumption, N (%)	489 (23.1%)	139(36.0%)	<0.001

^‡^General obesity: BMI≧25 kg/m^2^. ^§^Abdominal obesity: waist circumference of men ≧90 cm or waist circumference of women ≧80 cm*P value of independent t-test or Chi-square test. BMI, body mass index. WC, waist circumference. HDL-C, high density lipoprotein cholesterol. SBP, systolic blood pressure. DBP, diastolic blood pressure.

Table 2 shows demographic and clinical characteristics of normal, tubular, villous-rich adenoma groups. All variables including age, BMI, waist circumference, fasting glucose, TC, TG, HDL-C, SBP, DBP, and the proportion of males, general obesity, abdominal obesity, diabetes, hypertension, smoking and alcohol consumption showed significant differences among the three subgroups.

**Table 2:** Comparisons of demographic and clinical characteristics between normal, tubular, villous-rich^† ^adenoma groups

Variables	Normal (n = 2120)	Tubular adenoma (n = 333)	**Villous-rich**^**†**^**adenoma (n = 53)**	P * value
Age (years)	49.6 ± 12.4	55.5 ± 10.7	59.3 ± 11.3	<0.001
Male, N (%)	1239 (58.4%)	233 (70.0%)	39 (73.6%)	<0.001
BMI (kg/m^2^)	24.5 ± 3.4	25.2 ± 3.4	25.6 ± 3.7	0.016
WC (cm)	84.3 ± 10.4	87.3 ± 10.3	88.6 ± 11.0	<0.001
Fasting glucose (mg/dL)	93.3 ± 22.8	99.6 ± 27.2	109.1 ± 33.6	<0.001
Total cholesterol (mg/dL)	194.7 ± 36.9	199.9 ± 38.9	202.4 ± 36.4	0.024
Triglyceride(mg/dL)	129.4 ± 79.2	143.3 ± 81.6	163.2 ± 94.7	<0.001
HDL-C (mg/dL)	45.5 ± 11.8	43.9 ± 11.7	42.4 ± 12.0	0.012
SBP (mmHg)	117.4 ± 20.4	121.3 ± 16.6	130.9 ± 18.6	<0.001
DBP (mmHg)	68.8 ± 13.9	71.6 ± 9.7	74.2 ± 11.4	<0.001
General obesity^‡^, N (%)	869 (41.0%)	167 (50.2%)	27 (50.9%)	0.003
Abdominal obesity^§^, N (%)	894 (42.2%)	175 (52.6%)	27 (50.9%)	0.001
Hypertension, N (%)	478 (22.5%)	97 (29.1%)	27 (50.9%)	<0.001
Diabetes mellitus, N (%)	304 (14.3%)	80 (24.0%)	19 (35.8%)	<0.001
Smoking, N (%)	616 (29.1%)	125 (37.5%)	19 (35.8%)	0.005
Alcohol consumption, N (%)	489 (23.1%)	124 (37.2%)	15 (28.3%)	<0.001

^†^Villous-rich: tubulovillous and villous. ^‡^General obesity: BMI ≧25 kg/m^2^. ^§^Abdominal obesity: waist circumference of men ≧90 cm or waist circumference of women ≧80 cm*P value of ANOVA or Chi-square test. BMI, body mass index. WC, waist circumference. HDL-C, high density lipoprotein cholesterol. SBP, systolic blood pressure. DBP, diastolic blood pressure.

**Figure 1:**
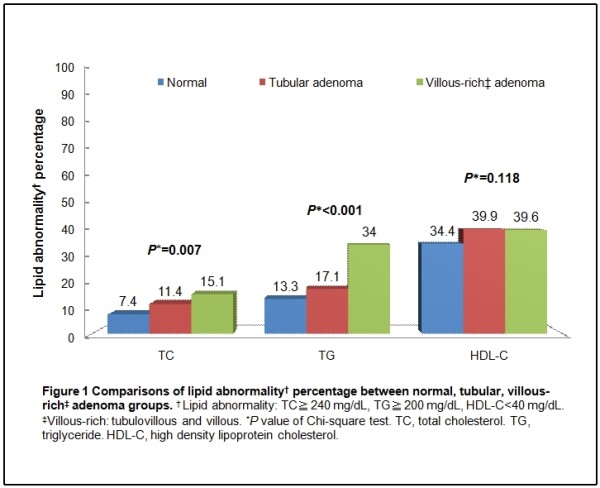
**Comparisons of lipid abnormality^† ^percentage between normal, tubular, villous-rich^‡ ^adenoma groups**. ¥ ^† ^Lipid abnormality: TC ≧240 mg/dL, TG ≧200 mg/dL, HDL-C < 40 mg/dL. ^‡^Villous-rich: tubulovillous and villous. *P value of Chi-square test. TC, total cholesterol. TG, triglyceride. HDL-C, high density lipoprotein cholesterol.

Figure 1 demonstrates the proportion of lipid abnormalities according to ATP III criteria among the three subgroups. There were significantly statistical differences among the three subgroups with regard to high TC (*P *= 0.007) and high TG (*P *< 0.001), but not with regard to low HDL-C (*P *= 0.118).

Table 3 shows the results of multinomial logistic regression analyses, and the OR and 95% CI of lipid profile for the risk of tubular adenoma and villous-rich adenoma. In Model 1, with adjustment for age and gender, high TC was related to both tubular (OR = 1.58, 95%CI: 1.08-2.32) and villous-rich adenoma (OR = 2.29, 95%CI: 1.05-5.01), and high TG was associated with the risk of villous-rich adenoma (OR = 3.35, 95%CI: 1.87-6.00), but not with tubular adenoma (OR = 1.35, 95%CI: 0.99-1.84). Further adjustment for general obesity, abdominal obesity, diabetes, hypertension, smoking, and alcohol consumption found that only high TG was associated with the risk of villous-rich adenoma (OR = 3.20, 95%CI: 1.71-6.01)(Model 2). Moreover, when taking the three lipid profiles into consideration together, high TG was still an independent correlation factor of villous-rich adenoma (OR = 4.42, 95%CI: 2.03-9.63) (Model 3).

**Table 3:** Multinomial logistic regression analysis for tubular adenoma and villous-rich^† ^adenoma in relation to lipid abnormalities

Lipid abnormality	**Model 1**^**‡**^		**Model 2**^**§**^		**Model 3**^**¶**^	
			
	Tubular	Villous-rich	Tubular	Villous-rich	Tubular	Villous-rich
Total cholesterol						
<240 mg/dL	1.00	1.00	1.00	1.00	1.00	1.00
≧240 mg/dL	1.58	2.29	1.44	1.93	1.54	0.63
95% CI	(1.08-2.32)	(1.05-5.01)	(0.97-2.13)	(0.86-4.35)	(0.83-2.84)	(0.23-1.69)
*P *value	0.019	0.038	0.074	0.111	0.168	0.356
Triglyceride						
<200 mg/dL	1.00	1.00	1.00	1.00	1.00	1.00
≧200 mg/dL	1.30	3.42	1.20	3.20	0.91	4.42
95% CI	(0.94-1.78)	(1.89-6.21)	(0.86-1.67)	(1.71-6.01)	(0.54-1.53)	(2.03-9.63)
*P *value	0.108	<0.001	0.278	<0.001	0.733	<0.001
HDL-C						
≧40 mg/dL	1.00	1.00	1.00	1.00	1.00	1.00
<40 mg/dL	1.10	1.04	1.08	0.92	1.04	0.72
95% CI	(0.86-1.42)	(0.58-1.86)	(0.83-1.40)	(0.50-1.67)	(0.80-1.36)	(0.38-1.34)
*P *value	0.453	0.889	0.573	0.774	0.769	0.301

^†^Villous-rich: tubulovillous and villous. ^‡^Model 1: multivariate odds ratio adjusted for age and gender. ^§^Model 2: multivariate odds ratio adjusted for age, gender, general obesity, abdominal obesity, hypertension, diabetes, smoking, and alcohol consumption. ^¶^Model 3: multivariate odds ratio adjusted for total cholesterol, triglyceride, HDL-C, and all the variables in Model 2. HDL-C, high density lipoprotein cholesterol. CI, confidence interval.

In further analyses of different gender, with adjustment for the same variables in Model 3, high TG was highly related to villous-rich adenoma in women (OR = 17.27, 95%CI: 4.40-67.75). There was a trend toward villous-rich adenoma in men with high TG (OR = 2.50, 95%CI: 0.90-6.96), although not reaching statistical significance (*p *= 0.078). In addition, when analyzing by age strata, with adjustment for the same variables in Model 3, high TG was related to villous-rich adenoma in both older subjects (OR = 4.03, 95%CI: 1.68-9.66) and in younger subjects (OR = 5.24, 95%CI: 0.95-28.90), although the later showed marginal statistical significance (p = 0.057) (tables not showed).

Because of limited subject numbers with villous-rich adenoma, we did not perform detailed cross-tabulated analyses of age and gender.

## Discussion

Our study showed that subjects with adenoma had higher TC and TG, and lower HDL-C levels than normal subjects. After considering more confounding factors, including not only age and gender, but also total cholesterol, HDL-C, general obesity, abdominal obesity, hypertension, diabetes, smoking, and alcohol consumption, only high TG was associated with the risk of tubulovillous/villous adenoma in rectosigmoid colon. We found that subjects with adenoma were older, and had higher fasting glucose and blood pressure than normal subjects. Male gender, diabetes, hypertension, general obesity, abdominal obesity, smoking, and alcohol consumption were also more common in those with adenoma. As described in previous studies, the older subjects had a higher risk of adenoma [23-27]. It has been suggested that somatic mutations accumulate with age and appear in adenoma, the precursor of colorectal cancer [4], but the mechanism for this still needs to be clarified in future research. With regard to gender differences, McCashland et al. suggested that estrogen may have a protective role in preventing adenoma formation via several mechanisms, such as estrogen receptor genes, decreased secondary bile acid, and decreased serum levels of insulin-like growth factors [28]. Therefore, men have a higher prevalence of colon polyps than women. On the other hand, diabetes [29,30], hypertension [31,32], general obesity [33,34], central obesity [35,36] are more common in subjects with adenoma. Insulin resistance or hyperinsulinemia might play an important role in the development of adenoma [24,29-36]. In addition, cigarette smoking has been consistently associated with a higher risk of colorectal adenoma [37,38] and a positive association of alcohol use with colorectal adenoma was found in some studies [39-41], but not in others [42,43]. Our study showed both smoking and alcohol consumption were positively correlated with the risk of rectosigmoid adenoma. The mechanisms are still not well understood, but carcinogens which are formed during the combustion of cigarettes seem likely to be involved in smoking's effect on adenoma.

The effect of serum lipids on colorectal adenoma was inconclusive in previous studies because of different methodologies and inadequate sample sizes [6-15]. Moreover, different cut-points of serum lipids were used in some studies, but we adopted the widely used ATP III criteria for our analysis. Subjects with adenoma had more lipid abnormalities than normal subjects in our research. In Taiwan, there were studies showing that low HDL-C and high TG in metabolic syndrome are associated with increased risk for colorectal adenoma [31,44]. However, they weren't aimed at the histological patterns of adenoma. Our study further investigated the association of serum lipids with different histological patterns of adenoma. Relatively few studies have attempted this in the past. Houghton et al. investigated 158 patients; among them, one in 10 (9.5%, 15/158) of subjects has polyps of villous histology. They found that cholesterol levels were positively associated with a greater likelihood of villous histology (OR, 1.18; 95%CI, 1.02-1.37) [17]. However, the significantly positive association between TC and villous adenoma disappeared after further adjusting for TG and HDL in our study. Another study by Tabuchi et al. performed a large scale retrospective study with 4,887 patients to analyze the correlation between the incidence of colorectal adenoma and serum levels of TC and TG. Multiple logistic regression analysis with adjustment for age and gender revealed that TG was an independent correlation factor in males with tubular adenoma, but not with villous adenoma [16]. Consequently, our adjusted models considered more confounding factors than Tabuchi's. The association of high TG with tubular adenoma was lost in our study; instead, its association with the risk of tubulovillous/villous adenoma appeared. One possible explanation is that, different to the previous studies, we had fewer subjects with pure villous adenoma and we utilized a different statistical methodology. More confounding factors were considered and entered into our final regression analysis. On the other hand, we had adopted ATP III criteria that were commonly used in clinical practice but were unusually applied for analysis in other studies. TC and HDL-C had no association with tubular or tubulovillous/villous adenoma, similar to the results of previous studies about cholesterol and overall adenoma [6,13,15].

There are several possible mechanisms that might explain our findings. First, hypertriglyceridemia is associated with hyperinsulinemia and insulin resistance [45-47]. Elevated insulin may inhibit apoptosis by interacting with insulin-like growth factors-I receptor, enhancing nuclear factor-κB activation or decreasing peroxisome proliferator-activated receptor-γ activation [24]. A lower rate of apoptosis in normal mucosa is an early event in the carcinogenesis process, and this may eventually develop into colorectal adenoma and even colorectal cancer. Second, patients with cholestyramine treatment and ileal exclusion suffer from the malabsorption of bile acids, which leads to a compensatory increase in bile acid biosynthesis, causing an increase in serum total triglycerides as a result of an increase in the VLDL fraction [48,49]. Increased bile acids are secreted into the bile and enter the intestine where they undergo further biotransformations and become secondary bile acids in the colon. Secondary bile acids are potent nonsubstrate inhibitors of glutathione sulfotransferase activity which is involved in the detoxification of exogenous carcinogens, and some of their potentially toxic biological activities might lead to mutagenicity, transforming activity, and DNA-strand breakage [50]. Deoxycholic acid, a secondary bile acid, was found to stimulate colorectal epithelial proliferation and promote adenoma formation [51]. As is well known, tubulovillous and villous adenoma have a higher potential for malignancy, and thus, we suggest that tubulovillous/villous adenoma might be more deeply affected by the above mentioned mechanisms.

There are several limitations to this study. First, it only includes subjects with colorectal adenoma in the rectosigmoid colon, not in all portions of the colon. In addition, the study's populations were all Taiwanese, and the findings might not be generalisable to other ethnic groups. Finally, our study is a cross-sectional one, and thus cannot provide enough temporal evidence of a causal relationship between serum lipids and the histological pattern of rectosigmoid adenoma. The strengths of the study, however, are that we strictly controlled various confounding factors to reveal the actual correlation, and adopted common ATP III criteria for analysis of serum lipids to provide practical information for primary care physicians.

## Conclusions

In our study, high serum triglyceride seems to be a strong independent correlation factor with tubulovillous and villous adenoma in the rectosigmoid colon. As a consequence, to encourage patients with hypertriglyceridemia to reduce their serum triglyceride level seems to be able to lower the risk of tubulovillous and villous adenoma in the colorectum, and thus might be one method to reduce the incidence of colorectal cancer.

## Competing interests

The authors declare that they have no competing interests.

## Authors' contributions

ZJS performed statistical analysis and drafted the manuscript while YHH, JSW, YCY, YFC, FHL and CJC participated in the revision and verification with critical insights and professional inputs. The tables and figure in this article were drafted by ZJS. All authors had substantial contributions to the collection, analysis and interpretation of data presented in the manuscript. All authors read and approved the final manuscript.

## Pre-publication history

The pre-publication history for this paper can be accessed here:

http://www.biomedcentral.com/1471-230X/11/54/prepub
